# Motility of Colonial Choanoflagellates and the Statistics of Aggregate Random Walkers

**DOI:** 10.1103/PhysRevLett.116.038102

**Published:** 2016-01-22

**Authors:** Julius B. Kirkegaard, Alan O. Marron, Raymond E. Goldstein

**Affiliations:** Department of Applied Mathematics and Theoretical Physics, Centre for Mathematical Sciences, University of Cambridge, Wilberforce Road, Cambridge CB3 0WA, United Kingdom

## Abstract

We illuminate the nature of the three-dimensional random walks of microorganisms composed of individual organisms adhered together. Such *aggregate random walkers* are typified by choanoflagellates, eukaryotes that are the closest living relatives of animals. In the colony-forming species *Salpingoeca rosetta* we show that the beating of each flagellum is stochastic and uncorrelated with others, and the vectorial sum of the flagellar propulsion manifests as stochastic helical swimming. A quantitative theory for these results is presented and species variability discussed.

Active microparticles, self-propelled by stored energy or that available from the environment, typically exhibit directed motility combined with rotational diffusion, leading to random walks that at large times are statistically similar to their equilibrium counterparts. For artificial swimmers such as Janus particles [1], powered by inhomogeneous surface chemical reactions, the source of randomness is the same thermal fluctuations that translate Brownian particles, but here rotate them [2]. In biology, several paradigms for stochastic locomotion exist. For single-celled organisms, stochastic beating leads to noisy swimming paths [3], and active processes such as flagellar bundling or unbundling by bacteria [4] and synchronization or desynchronization in algae [5] enhances this stochasticity. “Obligate” eukaryotic polyflagellates such as the ciliate *Paramecium* [6] and the alga *Volvox carteri* [7], exhibit large-scale flagellar coordination, and increased regularity of motion.

Here we study motility in an important example of a “facultative” colonial organism, the choanoflagellate *Salpingoeca rosetta* (Fig. 1), which exhibits uni- and multicellular forms with variable cell number. Single cells of *S. rosetta*, like other microorganisms, are random walkers (see the Supplemental Material [8]). We report three main experimental results: (i) individual flagella of the constituent cells beat stochastically, (ii) flagella on a given colony display negligible cross-correlation, and (iii) the swimming trajectories of colonies are stochastic helices. These results suggest a hitherto unrecognized class of microorganisms, here called aggregate random walkers (ARWs): those built by stitching together individual random walkers [9]. We construct a minimal model to explain this motility.

Choanoflagellates are the closest unicellular relatives of animals [10]. They filter feed by using their flagellum to drive fluid through an eponymous funnel-shaped collar [11]. This beating also confers motility. Figure 1 shows a rosette colony of *S. rosetta* which is held together by an extracellular matrix, filopodia, and intercellular bridges [12]. Colonies form by cell division, not aggregation [13]. The evolutionary advantage of the colonial form is not fully understood, but it is triggered by certain bacteria [12,14], and theory suggests that chainlike colonies have enhanced nutrient uptake [15].

*S. rosetta* (obtained from Dr. Barry Leadbeater, University of Birmingham, UK) were cultured in artificial seawater [36.5 g/L Marin Salts (Tropic Marin, Germany)]. To provide a food source for prey bacteria, organic enrichment [4 g/L Proteose Peptone (Sigma-Aldrich, USA), 0.8 g/L Yeast Extract (Fluka Biochemika)] was added to the cultures at 15 *μ*l/ml. Cultures were grown at 22 °C and split every 4–7 days. To study the flagella beat dynamics, colonies were stuck to poly-L-lysine (0.01%, Sigma) treated microscope slides and flagella beats imaged at 500 fps (Fastcam SA3, Photron, USA) in bright field. Image template matching was employed to track the motion of the only slightly moving colonies, and in the local frame of the organism, the bases of the flagella were tracked by techniques similar to that of the active contour model [16], yielding as a readout of beating the angle *θ*(*t*) defined in Fig. 1(b).

Figures 2(a) and 2(b) show *θ*(*t*) from two flagella on the same colony, and it is clear that they have distinct frequencies. In general, the beating frequencies *f*, found by Fourier transforming *θ*(*t*), show a surprisingly high variability [Fig. 2(e)]. The normalized autocorrelation *C*_*θ*_(Δ*t*) = ⟨ *θ* (*t*) θ (*t* + Δ*t*) ⟩ _*t*_ / *θ*(*t*) ^2^ ⟩ _*t*_ for a single flagellum is plotted in Fig. 2(c). Similar to the function discussed [3,17] in the context of flagellar beating in *Chlamydomonas*, the data are consistent with *C*_*θ*_ = exp (− |*t*| */τ*) cos (2*πft*), the envelope of which is shown in the figure. The decay time *τ* also shows a very high degree of variability [Fig. 2(f)], but all are < 1 s, suggesting high stochasticity. Within colonies, the cross correlation between flagella $C_{\theta ,\theta^{\prime }}(\text{Δ}t)={\langle \theta (t)\theta^{\prime }(t+\text{Δ}t)\rangle }_{t}/\sqrt{\langle \theta {\left(t\right)}^{2}\rangle_{t}\langle \theta^{\prime }{\left(t\right)}^{2}\rangle {\;}_{t}}$ [Fig. 2(d)] is negligible (only a very slight signal can be made out, which we attribute to the overall wiggling of the colony—see Supplemental Material [8], Video 1). All cross-correlation signals were found to be less than 0.05 [Fig. 2(g)]. The lack of correlation between beating flagella in colonies makes *S. rosetta* an ideal model organism for ARWs.

In studying the swimming trajectories of *S. rosetta*, ensemble averages taken over many colonies will eliminate features related to colony-specific morphology (cell location and flagella orientation). To overcome this lack of “ergodicity,” we obtained long tracks of 36 individual colonies. In-house software logged and synchronized the position of the *xy* stage (MS-2000, ASI, USA) to a camera (Imaging Source, Germany) filming in bright field at 15 fps. This enabled tracking of colonies moving in three dimensions at distances much longer than the field of view. To track the particles a Gaussian-mixture model [18] was applied to estimate the moving background and, subsequently, the tracks were manually controlled. Figure 3(a) shows three examples, all ~20 min in length. On close inspection [inset of Fig. 3(a)] we observe that the trajectories are noisy helices. The mean squared displacement ⟨**Δ***r*^2^⟩ = ⟨ [**x** (*s + t*) − **x** (*s*)] ^2^ ⟩_*s*_ [Fig. 3(b)], shows an early time ballistic ~*t*^2^ behavior (for *t <* 1 s) and late time diffusive ~*t* form (inset) similar to that of Janus particles [2]. However, comparing these curves to those of conventional active Brownian particles (see the Supplemental Material [8]), we observe a different intermediate time behavior. These bumps [Fig. 3(b)] appear precisely because some of the constituent cells may beat off center and induce internal (effective) torques producing stochastic helical trajectories. To highlight the underlying regularity of this helical swimming, we calculate the velocity autocorrelation *C*_*v*_(*t*) = ⟨ **v** (*s* + *t*) · **v** (*s*) *⟩*_*s*_ [Fig. 3(c)], which oscillates at the frequency of the induced rotation and decays on a time scale of several oscillation periods.

Active random walks have been the attention of much research [19,20], but only recently have rotational torques been incorporated. *External* torques appear on, e.g., magnetotactic bacteria in the presence of magnetic fields [21] and gyrotactic organisms such as certain algae in gravitational fields [22] and can be treated analytically [23]. However, the present *internal* torques can be treated analytically only in two dimensions [24] and numerical [25] or approximative [26] methods are needed in three dimensions. Below, we develop an approximate 3D theory with the goal of simple analytical functions that can be used to extract physical quantities and interpret the data.

The diffusion of a random walker can be described by the Langevin equation $d\text{x}(t)=\text{v}(t)dt+\sqrt{2D}d\text{W}(t)$, where *D* is the translational diffusion constant and ***W***(*t*) a standard vector Wiener process with ⟨ *d****W***_*i*_(*t*)*d****W***_*j*_(*t*^′^) ⟩ = *δ*_*ij*_*δ*(*t* – *t*^′^). The case ***v***(*t*) = **0** is a passive particle and leads to the projected mean squared displacement ⟨ Δ*r*^2^⟩ ≡ ⟨ Δ*x*^2^ + Δy^2^⟩ = 4*Dt*. Building an ARW from *passive* particles leads to no new behavior, but motile particles also have a stochastic velocity term. In the simplest case in two dimensions, the speed *v* is constant and ***v***(*t*) *v*(cos *θ*(*t*), sin *θ*(*t*)) evolves stochastically through *θ*(*t*). The choice $d\theta =\sqrt{2D_{r}}dW_{r}(t)$ leads to the conventional result $\text{Δ}r^{2}=\left(2v^{2}/D_{r}^{2}\right)\left(D_{r}t+e^{-D_{r}t}-1\right)+4Dt$, which behaves ballistically, ⟨**Δ***r*^2^ ~ *t*^2^⟩ ~ *t*^2^, at early times, but diffusively, ⟨**Δ***r*^2^*⟩* ~ *t*, at longer times (see the Supplemental Material [8]) with an enhanced diffusion constant *D*_∞_ = *D* + *v*^2^/2*D*_*r*_. Contrary to passive random walkers, active random walkers’effective diffusion constant can vary with dimension. The 3D result is *D*_∞_ = *D + v*^2^/3*D*_*r*_ for rotational diffusion around a random vector orthogonal to ***v*** and *D*_∞_ = *D + v*^2^/6*D*_*r*_ for diffusion around three orthogonal directions. Typically, *D* ≪ *v*^2^*/D*_*r*_ and passive diffusion can be ignored.

The Reynolds number for *S. rosetta* is Re ~ 10^−4^. At such low Reynolds numbers, inertia is negligible and the fluid dynamics becomes governed by the linear Stokes equation. Accordingly, self-propelled choanoflagellates are both force- and torque-free. We assume that *S. rosetta* are spherelike such that couplings between translations and rotations can be ignored. Heuristically then, the velocity of a colony ***v***(*t*) is approximately a linear sum of the velocities that the constituents would have had swimming independently, ***v***(*t*) ≈ *η* Σ ***v***_*i*_(*t*), the factor *η* accounting for the change in drag with the radius *a* of the colony, as *η* ~ *a*^−1^. If some of the walkers comprising the colony, placed at positions {r*_i_*}, beat off center, an angular velocity ***ω***(*t*) ≈ *η*_*r*_ Σ ***r***_*i*_ × ***v***_*i*_ will also be induced, where *η*_*r*_ ~ *a*^−3^. Since {***v***_*i*_} and {***r***_*i*_} are given in the local coordinate system of the particle, they must be rotated along with the particle. For a two dimensional ARW, this motion is described by ***v***(*t*) = *v*(*t*)(cos *θ*(*t*); sin *θ*(*t*)), where |*v*(*t*)| = | ***v***(*t*)|, $d\theta =\text{ω}(t)dt+\sqrt{2D_{r}}dW(t)$, ***ω***(*t*) = ±|**ω**(*t*)|, and *D*_*r*_ is an effective rotational diffusion constant which can be calculated if the individual stochastic processes are prescribed. With *v*(*t*) constant, constant *ω*(*t*) yields circles in the absence of noise. In three dimensions, such motion leads to helices, making (2D projected) three-dimensional ARWs behave very differently from 2D ones and necessitating a full 3D theory.

In three dimensions we let the velocity evolve according to *d****v***(*t*) = *d*Ω(*t*) ⊗ ***v***(*t*) ~ *d*Ω(*t*) × ***v***(*t*) − 2*D*_*r*_***v***(*t*)*dt*, where $d\text{Ω}(t)=\text{ω}(t)dt+\sqrt{2D_{r}}dW_{r}(t)$, ⊗ is the Stratonovich cross product and 2*D*_*r*_***v***(*t*) is the noise induced drift in the Itō interpretation, ensuring the correct magnitude of ***v***(*t*) (see the Supplemental Material [8]). With the goal of a minimal model, we take the swimming speed constant in the approximation to follow. Likewise, we have $d\text{ω}(t)=\sqrt{2D_{r}}dW_{r}(t)⊗\text{ω}(t)$, and, similarly, we will assume the magnitude of ***ω***(*t*) constant. The simultaneous update of the translation and rotational velocity makes the system analytically quite intractable and thus we shall seek an approximate solution. As motivation, consider the case *D*_*r*_ = 0 with specified initial conditions ***v***(0) = ***v***_0_, ***ω***(0) = ***ω***_0_. This system can be solved exactly to yield $x(t)=x_{0}+\{{\text{ω}}_{0}\left({\text{ω}}_{0}\cdot v_{0}\right){\text{ω}}_{0}t+{\text{ω}}_{0}\times \left(v_{0}\times {\text{ω}}_{0}\right)\;\sin\;\left({\text{ω}}_{0}t\right)+{\text{ω}}_{0}\left({\text{ω}}_{0}\times v_{0}\right)\left[1-\cos\left({\text{ω}}_{0}t\right)\right]\}/{\text{ω}}_{0}^{3}$, or

(1) $$\text{x}(t)={\int \;}_{0}^{t}\text{v}\left(t^{\prime }\right)dt^{\prime },\;\text{v}(t)=\text{R}\cdot \left(\begin{array}{c} v_{\text{ω}}\cos{\text{ω}}_{0}t\\ v_{\text{ω}}\sin{\text{ω}}_{0}t\\ v_{p} \end{array}\right),$$

where = *v*_*ω*_ |**ω**_0_ × ***v***_0_| */ω*_0_, *v*_*p*_ = **ω**_0_ · ***v***_0_/***ω***_0_, ***ω***_0_ = |**ω**_0_|, and ***R*** is some orthogonal matrix. Equation (1) describes a helix of radius *v*_*ω*_*/ω*_0_ and mean speed *v*_*p*_ (averaged over 2*π/ω*_0_). The form of (1) inspires an approximative solution in the presence of noise in which the deterministic helix parameters define a continuous-time random walk with helixlike steps, the matrix ***R*** becoming a stochastic matrix process. As an effective description we assume ***R***(*t*) = ***R***_*x*_ (*α*) · ***R***_*y*_ (*β*) · ***R***_*z*_ (*γ*), where the matrix factors are rotations around the *x, y, z* axes and *α, β, γ* are taken independent and identically distributed with $d\alpha =\sqrt{2D_{r}}dW_{\alpha }(t)$ , which strictly speaking is only valid at *t =*0. While the approach breaks *x*-*y* symmetry, the approximation makes the system much more manageable, and simulations show it to be an overall good approximation for the statistics of interest at early times.

In the stationary limit we find for the 2D projected result (see the Supplemental Material [8]):

(2) $$\begin{array}{l} \langle \text{v}(\text{Δ}t)\cdot \text{v}(0)\rangle =\frac{e^{-2D_{r}|\text{Δ}t|}}{6}[2v_{p}^{2}\left(1+e^{D_{r}|\text{Δ}t|}\right)\\ \;\;\;\;\;\;\;\;\;\;\;\;\;\;\;\;\;\;\;\;\;\;\;\;\;\;\;\;\;\;\;+v_{\text{ω}}^{2}\left(3+e^{-D_{r}|\text{Δ}t|}\right)\cos\left({\text{ω}}_{0}\text{Δ}t\right)]. \end{array}$$

The 2D-projected mean squared displacement becomes

(3) $$\begin{array}{l} \langle \text{Δ}r^{2}(t)\rangle =\frac{v_{p}^{2}e^{-2D_{r}t}}{6D_{r}^{2}}\left(1+4e^{D_{r}t}\right)+4D_{\infty }t-a_{0}\\ \;\;\;+v_{\text{ω}}^{2}e^{-2D_{r}t}\left(\frac{4D_{r}^{2}-{\text{ω}}_{0}^{2}}{{\left(4D_{r}^{2}+{\text{ω}}_{0}^{2}\right)}^{2}}+\frac{\left(9D_{r}^{2}-{\text{ω}}_{0}^{2}\right)e^{-D_{r}t}}{3{\left(9D_{r}^{2}+{\text{ω}}_{0}^{2}\right)}^{2}}\right)\cos{\text{ω}}_{0}t\\ \;\;\;\;\;\;\;-v_{\text{ω}}^{2}e^{-2D_{r}t}\left(\frac{4{\text{ω}}_{0}D_{r}}{{\left(4D_{r}^{2}+{\text{ω}}_{0}^{2}\right)}^{2}}+\frac{2{\text{ω}}_{0}D_{r}e^{-D_{r}t}}{{\left(9D_{r}^{2}+{\text{ω}}_{0}^{2}\right)}^{2}}\right)\sin{\text{ω}}_{0}t, \end{array}$$

where the constant *a*_0_ enforces ⟨**Δ***r*^2^⟩ (*t* = 0) = 0. As *t* → ∞ we obtain ⟨**Δ***r*^2^⟩ = 4*D*_∞_*t*, where

(4) $$D_{\infty }=\frac{v_{p}^{2}}{4D_{r}}+\frac{v_{\text{ω}}^{2}D_{r}}{4}\left(\frac{1}{9D_{r}^{2}+{\text{ω}}_{0}^{2}}+\frac{2}{4D_{r}^{2}+{\text{ω}}_{0}^{2}}\right).$$

These results have been verified by simulations using the Euler-Maruyama method. It has previously been shown that reciprocal swimming enhances diffusion [20], and the last terms of Eq. (4), which are major contributions to the diffusion constant, embody this phenomenon.

Equations (2) and (3) describe the approximate functions corresponding to the data of Figs. 3(c) and 3(b), respectively. The diffusion constant *D*_∞_ can be extracted from the linear late-time behavior of ⟨**Δ***r*^2^⟩ [dashed gray in inset of Fig. 3(b)], and can be used in Eq. (4) to fix one of the model parameters in terms of the others. The remaining three are fitted simultaneously to the curves of Figs. 3(b) and 3(c). The experimental data are well described by the model as shown by the dashed lines in the figures. The relative magnitudes of the extracted velocities, *v*_*p*_ and *v*_*ω*_, reveal how much energy the organisms spent on effective (*v*_*p*_) and circular (*v*_*ω*_) swimming, for example, the blue curve in Fig. 3 has *v*_*p*_ = 11.4 and *v*_*ω*_ =13.6 *μ*m/s. While not producing the precise morphologies of the colonies, the fitted velocities combined with the extracted frequency ***ω***_0_, do constrain the possible configurations. Using the fitted velocities and a colony radius *a* ~ 5 *μ*m, we find an effective translational force of ~1 pN, and using ***ω***_0_, an effective torque ~4 pN · *μ*m: the small residual forces that propel and rotate a colony are on the order of that of a single cell.

Just as flagella beating in *S. rosetta* varies between cells, morphology varies between colonies as a result of the cell division process [13]. This stochasticity enables two colonies of similar size to swim very differently. To quantify this, we used in-house software to track ~750 colonies of varying size swimming in quasi-two dimensions between two cover slips, and when a colony was in focus the area of an ellipse fitted to its outline served as an estimator of size (see Supplemental Material [8], video 2). This method, while introducing uncertainty in area, enables high throughput. The speed of these versus size is shown in Fig. 4. To obtain model parameters, long tracks are needed. The parameters for 36 such tracks are given in the Supplemental Material [8], and the speed $\sqrt{v_{p}^{2}+v_{\text{ω}}^{2}}$ of those tracks is shown in Fig. 4 as green circles. There is a slow increase in speed with colony size. This trend can be explained by simple *ad hoc* models such as random orientation of cells in a spherelike structure: drag scales linearly with radius *a* but maximum propulsive force (the case where all propulsive forces point in one direction) scales like *a*^2^. However, there is an intriguing lack of very slow swimmers which would be predicted by such a model. Indeed, giving cells an orientation more parallel with its location would only yield slower swimming speeds. More importantly, Fig. 4 shows just how different colonies of similar size are: the stochastic processes underlying colony formation have high variances. From fits of the long tracks this stochasticity seems to apply to all model parameters (Supplemental Material [8]). This is contrary to, e.g., bacterial clumps where rotation rate clearly decreases with size [9]. Contrary to the phototactic response of *Chlamydomonas* and *Volvox*, in which the time scale of rotation is matched to inner chemistry [27], or the chemotactic response of sperm cells in which curvature and torsion of swimming paths are directly manipulated by the single beating flagellum [26], due to this stochastic morphology of *S. rosetta*, knowledge of the overall colony morphology and motion (e.g., ***ω***_0_) is arguably not available at the single-cell level, rendering “deterministic” chemotactic strategies difficult. Thus, one of the most important issues is the possibility of chemotaxis in aggregate random walks through suitable modulation of the independent constituents [28].

A fundamental operation in the theory of stochastic processes is their summation to yield a single effective process. The corresponding operation for random walkers, “stitching” them together, yields ARWs. As we have shown, there is a crucial complexity for random walkers: the underlying flagellar beating can also yield rotations, so the “summation rules” differ. Our results suggest that for simple random walkers the ARWs can be described approximately through four numbers: *v*_*ω*_, *v*_*p*_, *ω*_0_, and *D*_*r*_. The question of the correct summation rules for general random walkers (e.g., anisotropic, hydrodynamically translation-rotation coupled) remains open. Likewise, the transition, via, e.g., self-assembly or flagella growth, from high to low stochasticity in ARWs with nonindependent constituents is intriguing. The present exemplar, *S. rosetta*, is a very good approximation to what one might call an ideal biological ARW: independent constituents and a roughly spherical shape. Its mode of swimming raises many interesting questions about the evolution of multicellularity and on the nature and origin of noise, both internal and environmental.

We thank M. E. Cates, E. Lauga, K. C. Leptos, and T. J. Pedley for discussions, and an anonymous referee for insightful comments. Work supported by the EPSRC and St. Johns College, Cambridge (J. B. K.), ERC Advanced Investigator Grant No. 247333, and a Wellcome Trust Senior Investigator Award.

## Supplementary Material

SI

## Figures and Tables

**Fig. 1:**
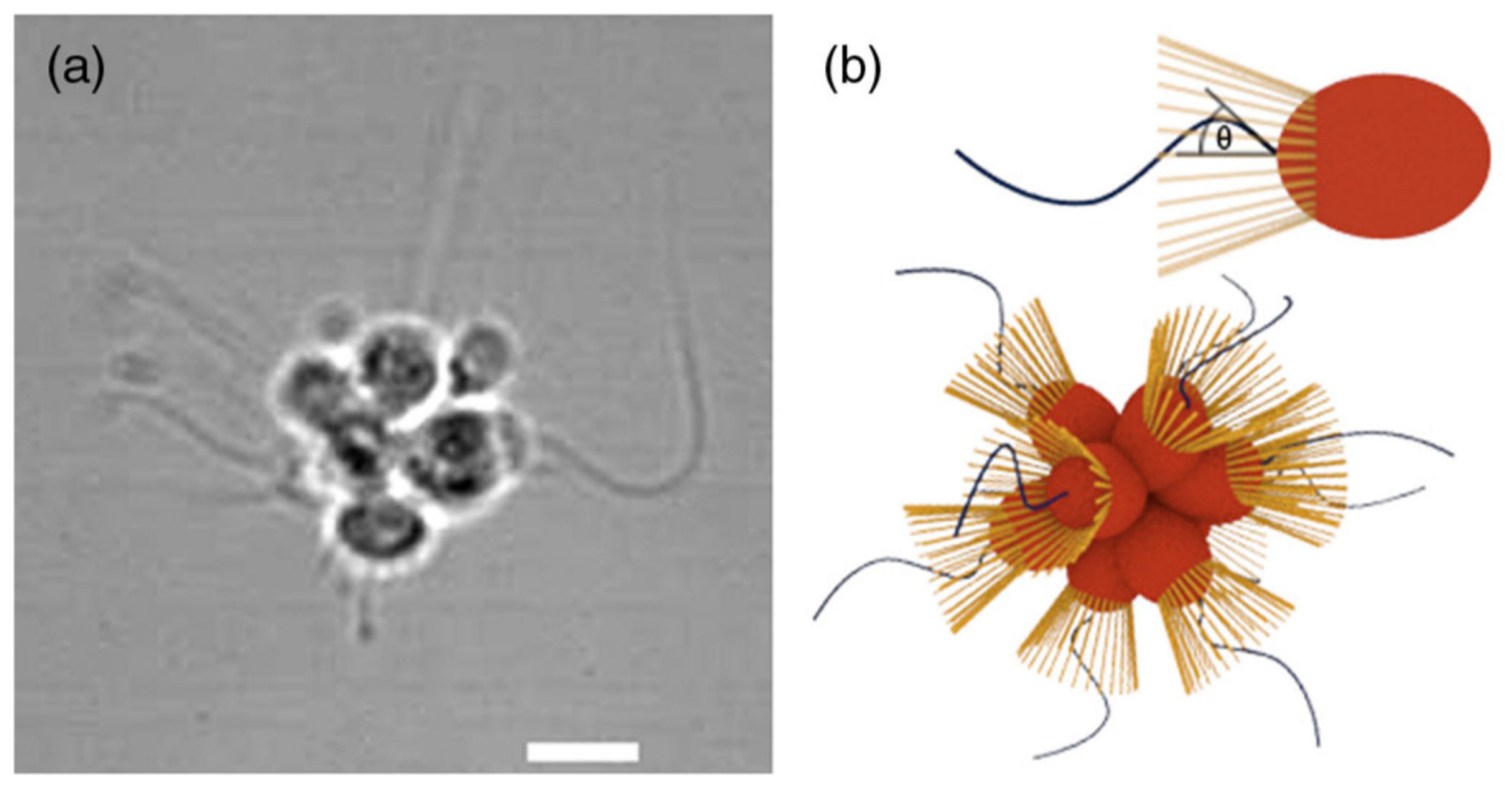
The choanoflagellate *S. rosetta*. (a) Bright field image (5 *μ*m scale) and (b) schematics of ‘slow-swimmer’ single cell, base angle *θ*, and rosette colony.

**Fig. 2:**
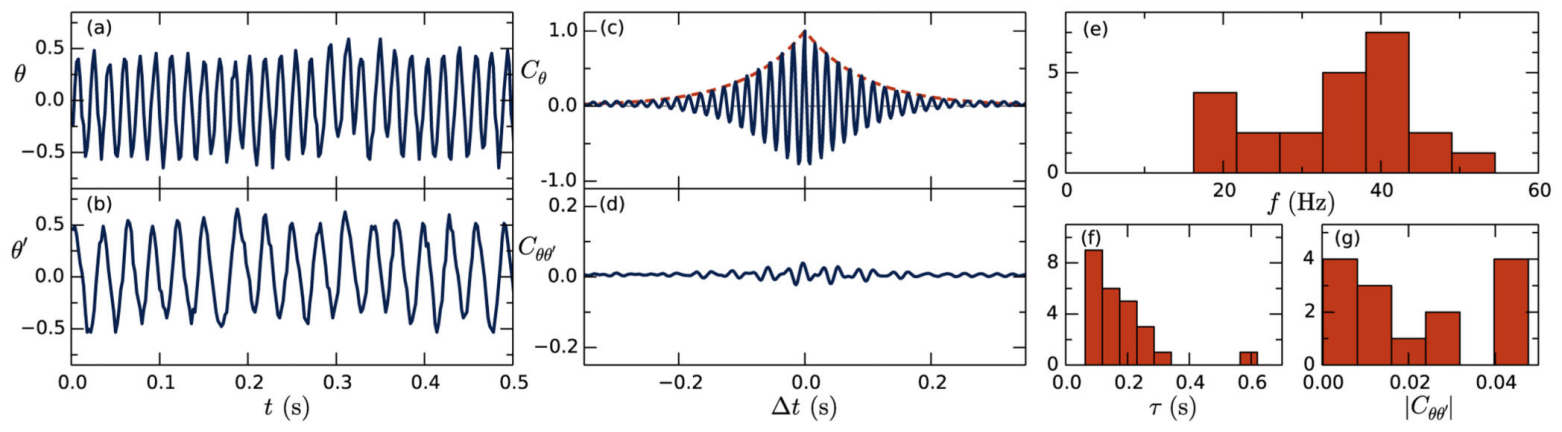
Flagellar beating dynamics. (a,b) Time series of the base angle *θ*(*t*) on two flagella within a single colony. (c) Autocorrelation function of *θ* for one flagellum, with fit of the envelope to an exponential decay (dashed red). (d) Cross correlation of *θ* between two flagella on the same colony. (e) Peak frequencies of *n* = 23 tracked flagella. (f) Decay time of autocorrelation in single flagella. (g) Magnitude of cross correlations between flagella in same colonies.

**Fig. 3:**
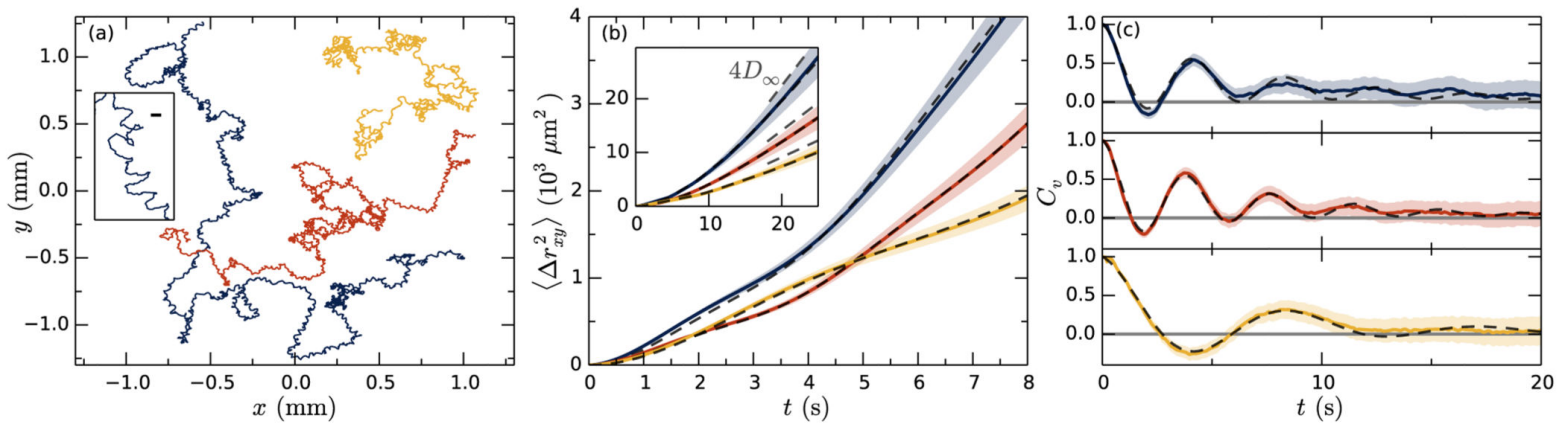
Random walks. (a) Long tracks of swimming *S. rosetta*. Inset scale bar is 10 *μ*m. (b) Projected mean squared displacement for individual walks (solid, error shaded) and fits of model (dashed). Inset shows a zoom-out with late-time linear behavior ~4*D*_∞_*t* (dashed, gray). (c) Velocity autocorrelation (solid, error shaded) and model (dashed) with parameters as in (b). Fitted parameters (red curve): ***ω***_0_ = 1.63 s^−1^, *v*_*p*_ = 9.0 *μ*m/s, *v*_*ω*_ = 12.1 *μ*m/s, *D*_*r*_ = 0.09 s^−1^.

**Fig. 4:**
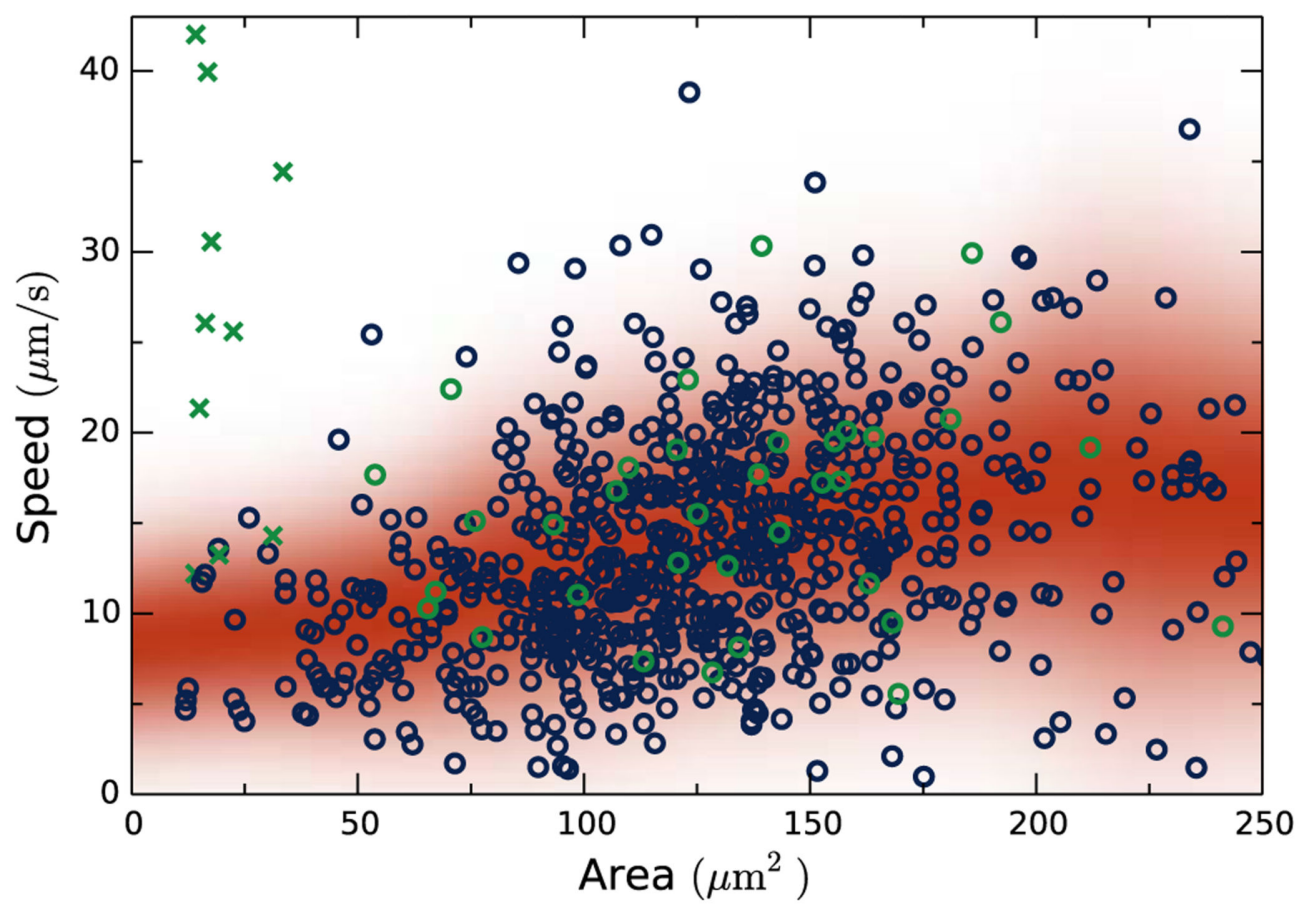
Speed vs size for ~750 colonies. Colony size is estimated by median *xy*-projected area. Colonies are blue and green circles, green speed being $\sqrt{v_{p}^{2}+v_{\text{ω}}^{2}}$ from model fits to the long tracks. Green crosses are single-celled fast swimmers [12]. Colored background indicates running mean and standard deviation.
