# Supplementary material for: Motility of Colonial Choanoflagellates and the Statistics of Aggregate Random Walkers
Source: Phys Rev Lett. Author manuscript; Available in PMC 2024 Jun 10. (PMC7616083; doi:10.1103/PhysRevLett.116.038102)
Supplement: SI [file EMS196590-supplement-SI.pdf]

Supplemental Information

Motility of Colonial Choanoflagellates
and the Statistics of Aggregate Random Walkers

Julius B. Kirkegaard, Alan O. Marron, Raymond E. Goldstein

Single cells

So-called 'slow-swimmer' *S. rosetta* unicells, similar in morphology to the individual cells that comprise a colony, clearly exhibit random walk behaviour. Fig. 1 shows the mean squared displacement of 32 *S. rosetta* slow-swimmers, each filmed for ∼1.5 minutes. The behaviour is well-described by the equation for conventional active random walkers,

$$\langle \Delta r^2 \rangle = (2v^2/D_r^2)\left(D_r t + e^{-D_r t} - 1\right). \tag{1}$$

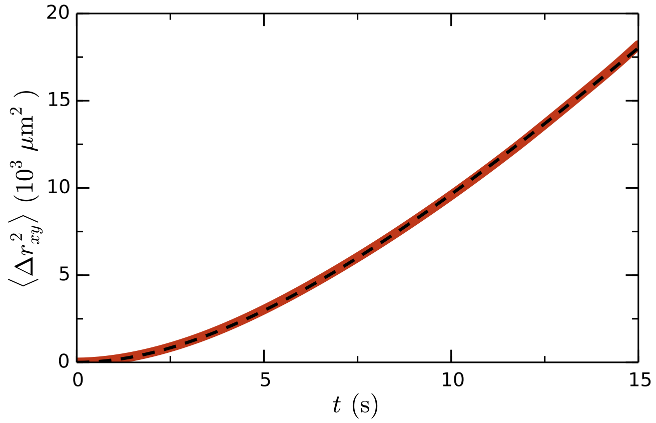

Figure 1: Squared distance moved averaged over 32 *S. rosetta* single cells. Overlayed fit (dashed) is to Eq. (1). Parameters: $v = 12.3$ μm/s, $D_r = 0.15$ s^{-1}.

The *active* rotational diffusion constants for both single cells and colonies (main text) are on the order of 0.1 s^{-1}. With a beat frequency $f \sim 40$ Hz, this corresponds to a distribution of angular deviations per beat with standard deviation $\sim\sqrt{D_r/f} = 3°$. The thermal rotational diffusion constant $D_r^{\text{thermal}} = k_B T/8\pi\mu a^3$ ranges from 0.012 to 0.0014 s^{-1} for radii 2.5 to 5.0 μm, at least an order magnitude below the active one.

Noise induced drift

The Langevin equations

$$\mathrm{d}\boldsymbol{v}(t) = \boldsymbol{\omega}(t) \times \boldsymbol{v}(t)\,\mathrm{d}t + \sqrt{2D_r}\,\mathrm{d}\mathbf{W}_r(t) \otimes \boldsymbol{v}(t) \tag{2}$$

$$\mathrm{d}\boldsymbol{\omega}(t) = \sqrt{2D_r}\,\mathrm{d}\mathbf{W}_r(t) \otimes \boldsymbol{\omega}(t) \tag{3}$$

must be interpreted in the Stratonovich sense for the magnitude of v and $\boldsymbol{\omega}$ to not grow indefinitely. Using $\mathrm{d}\mathbf{W}_r(t) = (\mathrm{d}W_1(t), \mathrm{d}W_2(t), \mathrm{d}W_3(t))$, Eq. (3) can be written as

$$\mathrm{d}\boldsymbol{\omega}(t) = \sqrt{2D_r} \begin{pmatrix} 0 & \omega_3(t) & -\omega_2(t) \\ -\omega_3(t) & 0 & \omega_1(t) \\ \omega_2(t) & -\omega_1(t) & 0 \end{pmatrix} \circ \begin{pmatrix} \mathrm{d}W_1(t) \\ \mathrm{d}W_2(t) \\ \mathrm{d}W_3(t) \end{pmatrix} \equiv \boldsymbol{\sigma}(t) \circ \begin{pmatrix} \mathrm{d}W_1(t) \\ \mathrm{d}W_2(t) \\ \mathrm{d}W_3(t)\,, \end{pmatrix}. \tag{4}$$

and likewise for Eq. (2). The corresponding Itō equation becomes

$$\mathrm{d}\boldsymbol{\omega}(t) = \boldsymbol{\sigma}(t) \cdot \begin{pmatrix} \mathrm{d}W_1(t) \\ \mathrm{d}W_2(t) \\ \mathrm{d}W_3(t) \end{pmatrix} + \frac{1}{2}\left((\boldsymbol{\sigma}(t)\cdot\nabla_\omega)^T\,\boldsymbol{\sigma}(t)\right)^T \mathrm{d}t \tag{5}$$

where T denotes transpose. The last term is the noise-induced drift, evaluating to

$$\frac{1}{2}\left[(\boldsymbol{\sigma}(t)\cdot\nabla_\omega)^T\,\boldsymbol{\sigma}(t)\right]_i = \frac{1}{2}\sum_{k=1}^{3}\sum_{j=1}^{3}\sigma_{kj}\,\frac{\partial\sigma_{ij}}{\partial\omega_k} = -2D_r\omega_i, \tag{6}$$

The calculation of $\mathbf{v}(t)$ follows the same procedure, and yields $-2D_r v_i$.

Derivation of random walker functions

Since the rotation angles α, β, and γ are Markov processes we can write the probability distribution functions as e.g. $P(\alpha(t')) = N(\alpha_0, 2D_r\, t')$ and $P(\alpha(t)|\alpha(t')) = N(\alpha(t'),$ $2D_r\,(t-t'))$ for $t' < t$, where $N(\mu, \sigma^2)$ is the normal distribution. Using $P(\alpha(t), \alpha(t'))$ $= P(\alpha(t)|\alpha(t'))P(\alpha(t'))$ we obtain averages such as

$$\begin{aligned} \langle \cos\alpha(t)\cos\alpha(t')\rangle &= \int_{-\infty}^{\infty}\mathrm{d}x\int_{-\infty}^{\infty}\mathrm{d}y\,\cos(x)\cos(y) \\ &\quad\times N_x(\alpha_0, 2D_r\min(t,t')|)N_y(x, 2D_r|t-t'|) \\ &= \frac{1}{2}e^{-D_r|t-t'|}\left(1+\cos(2\alpha_0)e^{-4D_r\min(t,t')}\right), \end{aligned} \tag{7}$$

which in the stationary limit can be used to find the velocity autocorrelations, e.g.

$$\begin{aligned} \langle v_x(t)v_x(s)\rangle &= \Big\langle \big(v_\omega\cos(\beta(t))\cos(\gamma(t))\cos(\omega_0 t) + v_p\sin(\beta(t)) \\ &\quad - v_\omega\cos(\beta(t))\sin(\gamma(t))\sin(\omega_0 t)\big) \times \big(v_p\sin(\beta(s)) \\ &\quad + v_\omega\cos(\beta(s))\cos(\gamma(s))\cos(\omega_0 s) - v_\omega\cos(\beta(s))\sin(\gamma(s))\sin(\omega_0 s)\big)\Big\rangle \\ &= \frac{1}{2}\,v_p^2\,e^{-D_r|t-s|} + \frac{1}{4}\,v_\omega^2 e^{-2D_r|t-s|}\,\cos(\omega_0(t-s)). \end{aligned} \tag{8}$$

The function only depends on the time difference $t-s$, which is the case for stationary autocorrelations (this is the very definition of a *weakly* stationary process). From

$$\begin{aligned} v_y(t) &= -v_p\sin(\alpha(t))\cos(\beta(t)) + v_w\big(\big[\sin(\alpha(t))\sin(\beta(t))\cos(\gamma(t)) \\ &\quad + \cos(\alpha(t))\sin(\gamma(t))\big]\cos(\omega_0 t) + \big[\cos(\alpha(t))\cos(\gamma(t)) \\ &\quad - \sin(\alpha(t))\sin(\beta(t))\sin(\gamma(t))\big]\sin(\omega_0 t)\big) \end{aligned} \tag{9}$$

we find in a similar manner

$$\langle v_y(t)v_y(s)\rangle = \frac{1}{4}\,v_p^2\,e^{-2D_r|t-s|} + \frac{1}{8}\,v_\omega^2\left(e^{-3D_r|t-s|} + 2e^{-2D_r|t-s|}\right)\,\cos(\omega_0(t-s)), \tag{10}$$

and the same for $\langle v_z(t)v_z(s)\rangle$. We project on to a random 2D plane by summing the x, y, z results followed by multiplication of $2/3$ (this gives a different result than simply summing the x and y components due to the asymmetry induced by the approximation). We thus find

$$\langle \boldsymbol{v}(\Delta t)\cdot \boldsymbol{v}(0)\rangle = \frac{e^{-2D_r|\Delta t|}}{6}\left[2v_p^2\left(1+e^{D_r|\Delta t|}\right)+v_\omega^2\left(3+e^{-D_r|\Delta t|}\right)\cos(\omega_0\Delta t)\right]. \quad (11)$$

The mean squared displacement is obtained by integrating the autocorrelation twice

$$\begin{aligned}\langle \Delta r^2(t)\rangle &= \int_0^t\int_0^t \langle \boldsymbol{v}(t')\cdot \boldsymbol{v}(t'')\rangle\,\mathrm{d}t'\mathrm{d}t'' \quad (12)\\ &= \frac{v_p^2 e^{-2D_r t}}{6D_r^2}\left(1+4e^{D_r t}\right)+4D_\infty t - a_0\\ &\quad + v_\omega^2 e^{-2D_r t}\left(\frac{4D_r^2-\omega_0^2}{(4D_r^2+\omega_0^2)^2}+\frac{9D_r^2-\omega_0^2}{3(9D_r^2+\omega_0^2)^2}e^{-D_r t}\right)\cos\omega_0 t\\ &\quad - v_\omega^2 e^{-2D_r t}\left(\frac{4\omega_0 D_r}{(4D_r^2+\omega_0^2)^2}+\frac{2\omega_0 D_r}{(9D_r^2+\omega_0^2)^2}e^{-D_r t}\right)\sin\omega_0 t,\end{aligned}$$

where

$$a_0 = \frac{5v_p^2}{6D_r^2}+v_\omega^2\left(\frac{4D_r^2-\omega_0^2}{(4D_r^2+\omega_0^2)^2}+\frac{9D_r^2-\omega_0^2}{3(9D_r^2+\omega_0^2)^2}\right). \quad (13)$$

As $t\to\infty$, $\langle \Delta r^2\rangle \sim 4D_\infty t$, where

$$D_\infty = \lim_{t\to\infty}\frac{\langle \Delta r^2\rangle}{4t} = \frac{v_p^2}{4D_r}+\frac{v_\omega^2 D_r}{4}\left(\frac{1}{9D_r^2+\omega_0^2}+\frac{2}{4D_r^2+\omega_0^2}\right). \quad (14)$$

The existence of the above (non-zero) limit confirms the diffusive behaviour.

Comparison of fit parameters

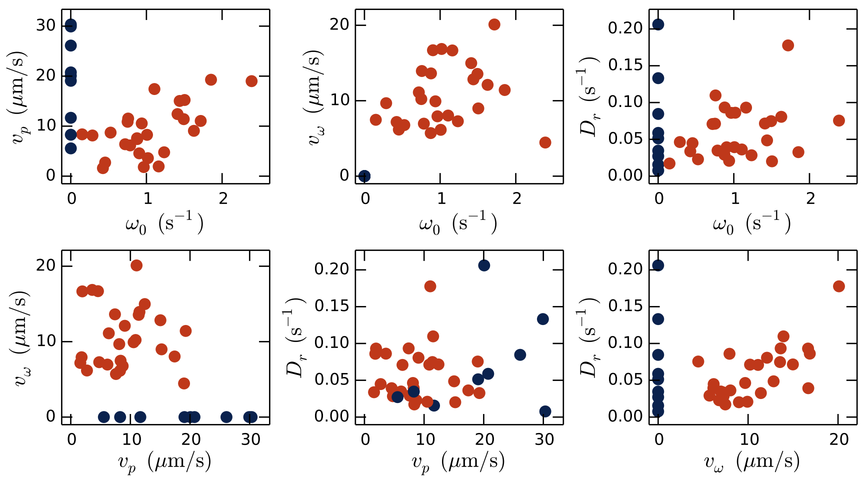

Figure 2: Comparison of fit parameters of 36 tracks. Tracks where ω_0, v_ω could be determined in red and tracks where ω_0, v_ω are forced to zero in blue.

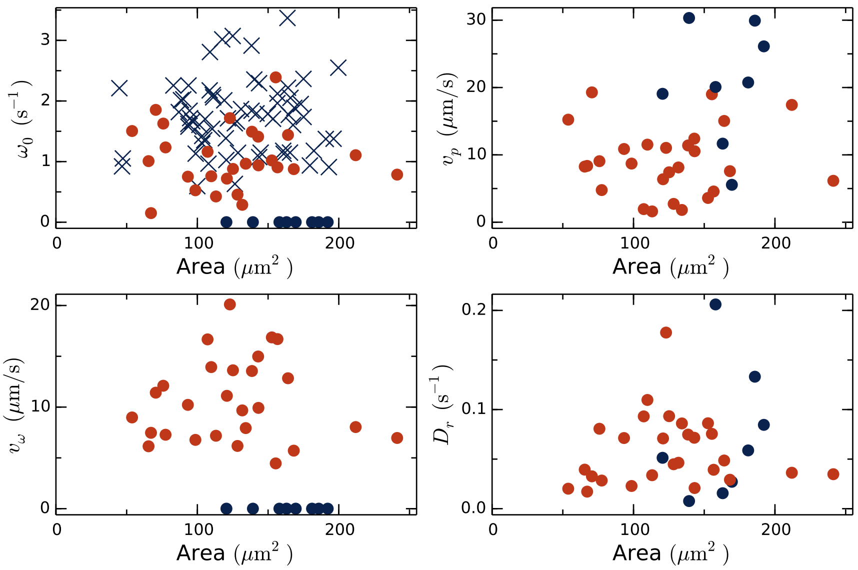

Figure 3: Comparison of fit parameters of 36 tracks to size of colony. Tracks where ω_0, v_ω could be determined in red and tracks where ω_0, v_ω are forced to zero in blue. ω_0 plot additionally includes estimates from the short track data in blue crosses.

Fig. 2 shows scatter plots of fit parameters of the model to 36 different *S. rosetta* colonies, indicating the high variances of all parameters. We note, however, that the determination of some parameters is difficult in certain regions. For instance, ω_0 and v_ω are hard to determine when either one becomes small, and accordingly we have forced them to zero in these cases and plotted them in blue. Naturally, these cases will have a higher v_p as is clear in the two plots in the left part of Fig. 2.

Applying the same area estimator as in Fig. 4 of the main text, the parameters can also be plotted as a function of size. Just as with swimming speed, Fig. 3 shows that the model parameters have very high variances and no clear dependence on size. For a subset of the short tracks we were able to fit the model well enough to estimate ω_0 and these are shown as blue crosses. However, the short track colonies for which good estimates could be obtained are biased towards high ω_0 (and v_ω). Nonetheless, there is no clear tendency for larger colonies to rotate slower as is the case for e.g. bacterial clumps [1]. For an interesting example of a big fast-spinning colony see the end of Supplemental Video 2, in which a colony has formed a dumbbell shape.

References

[1] J. Schwarz-Linek, C. Valeriani, A. Cacciuto, M.E. Cates, D. Marenduzzo, A.N. Morozov, and W.C.K. Poon, Phase separation and rotor self-assembly in active particle suspensions, Proc. Natl. Acad. Sci. USA **109**, 4052-4057 (2012).
